# Achieving the impossible: a review of magic-based interventions and their effects on wellbeing

**DOI:** 10.7717/peerj.6081

**Published:** 2018-12-06

**Authors:** Richard Wiseman, Caroline Watt

**Affiliations:** 1 Psychology, University of Hertfordshire, Hatfield, United Kingdom; 2 Psychology, University of Edinburgh, Edinburgh, United Kingdom

**Keywords:** Psychology, Occupational therapy, Magic tricks, Health, Intervention, Performing arts

## Abstract

Research has demonstrated that involvement with mainstream performing arts, such as music and dance, can boost wellbeing. This article extends this work by reviewing little-known research on whether learning magic tricks can have an equally beneficial effect. We first present an historic overview of several magic-based interventions created by magicians, psychologists and occupational therapists. We then identify the potential benefits of such interventions, and review studies that have attempted to systematically assess these interventions. The studies have mostly revealed beneficial outcomes, but much of the work is of poor methodological quality (involving small numbers of participants and no control group), and has tended to focus on clinical populations. Finally, we present guidelines for future research in the area, emphasizing the need for more systematic and better-controlled studies.

## Introduction

Research has shown that involvement with the performing arts can help boost psychological and physical wellbeing (for reviews see Fraser, Bungay & Munn-Giddings, 2014; Noice, Noice & Kramer, 2014; Stickley et al., 2017). This work has employed a variety of research designs (including randomised controlled trials, case studies, and observational designs) and tackled a wide variety of topics (including mental health issues, dementia and Parkinson’s disease, chronic illnesses, head injuries, substance abuse, and physical and developmental disabilities). Although most of this work has focused on mainstream forms of performance (such as acting, music, and dance), a small amount of the research has examined the role that conjuring can play within both health-care and educational settings.

This work has involved two main approaches.

First, some of the research has examined the benefits associated with clinicians and educators performing magic tricks. For example, within health-care settings, medics and therapists have described performing magic tricks to help reinforce positive health messages (Falkner, 1971; Lustig, 1994), reduce patient anxiety (Zhang et al., 2017), establish rapport during psychotherapy (Moskowitz, 1973; Howard, 1977; Spruill & Poidevant, 1993; Gilroy, 1998; Gilroy, 2001), deliver life lessons (Bowman, 1986; Bowman, 2004), and gain the co-operation of patients (Bow, 1988; Schwartz, 2003; Peretz & Gluck, 2005). Similarly, within a pedagogic context, educators have performed magic tricks to help boost student’s critical thinking skills (McCormack, 1985; Siegelman, 1985), promote interest in STEM topics (Papalaskari et al., 2006; Papalaskari et al., 2007) and communicate specific concepts in physics (Ruiz, 2006), chemistry (Haub, 2001), organisational behaviour (Krell & Dobson, 1999), computer science (Curzon & McOwan, 2008), statistics (Lesser & Glickman, 2009), mathematics (Simonson & Holm, 2003; Yew, 2005) and psychology (Solomon, 1980).

A second strand of magic-based research has taken a more participatory approach and focused on the potential benefits associated with learning how to perform conjuring tricks. This work has tended to appear in specialist academic journals and publications produced by the magic community. This paper reviews this little-known literature, presenting an historical overview of the area, identifying the claimed benefits of magic-based interventions, and reviewing work that has assessed these claims.

### Search strategy

Literature relating to learning magic tricks and wellbeing was identified by (i) entering the search terms ‘magic therapy’ and ‘magical therapy’ into databases from both academia and the magic community (including Google Scholar, Ovid Medline, Scopus and ‘Ask Alexander’ from the Conjuring Arts Research Centre), (ii) drawing on existing knowledge and personal contacts within academia and magic (namely David Britland, Will Houstoun, Sadie Broome, Alan McCormack, Richard Kaufman and Kevin Spencer), (iii) searching key books and articles that explore the relationship between magic, psychology and science (including Lamont & Wiseman, 2005; Macknik, Martinez-Conde & Blakeslee, 2010; Rensink & Kuhn, 2015; Lam, Lam & Chawla, 2017), (iv) examining the material referenced in located papers and books, and (v) contacting the authors of this material for additional information. All searches were undertaken in September 2018.

## Historical Overview

Magic-based interventions have been developed within both health and educational contexts. Each area will be discussed in turn.

### Health

Early work exploring the possible relationship between wellbeing and performing magic tricks appeared during the First World War. In 1915, conjurer and illustrator Charles Folkard wrote ‘Tricks For The Trenches And Wards’, a book that encouraged convalescing soldiers to perform magic tricks as a form of entertainment and rehabilitation (Draklof, 1915). At the start of the book Folkard described being inspired by a nurse commenting on the therapeutic value of magic, and in the remainder of the text presented a series of tricks, most of which can be performed with the hands alone. The magic literature contains a well-known anecdote from this era supporting the use of magic as occupational therapy. ‘Cardini’, one of the world’s most highly skilled magicians, initially learned how to manipulate playing cards whilst recuperating from shell shock after being wounded in the battle of the Somme. Later reflecting on his time in hospital, Cardini remarked: ‘Of course they [medics] didn’t know so much about occupational therapy back in the first World War, but manipulating my fingers with cards amounted to just that…’ (Beaumont, 1948).

During the Second World War, magician and psychiatrist Douglas Kelley published a lengthy article describing how he had successfully used magic tricks as a form of occupational therapy at the New York State Psychiatric Institute and Hospital (Kelley, 1940). An experienced and well-regarded magician, Kelley discussed why magic tricks are especially well suited to this type of work, noting, for instance, that they often only require a small amount of practice, can be easily adapted to suit the patient and require very little financial outlay.

Since then, several health practitioners have reported used magic-based interventions in their work. In addition, the following larger-scale initiatives have been launched.

In the late 1950s, a small group of American magicians established the ‘National Committee for Therapy Through Magic’, and encouraged performers to team up with medics and to teach magic tricks to their patients (Lopez, 1957). The initiative involved several well-known medical institutions, including the Mayo Clinic (Minnesota), the Bellevue Hospital (New York), and the Variety Children’s Hospital (Miami). Although the program received considerable coverage in the magical literature (see, e.g., Sibley, 1957; Christopher, 1958), it’s unclear how many magicians were actively involved and the initiative seems to have disappeared by the early 1960s.

In 1981, internationally renowned magician David Copperfield worked with Occupational Therapist Julie DeJean to create ‘Project Magic’. This program aimed to teach patients magic tricks to enhance their wellbeing, motivation, and self-esteem. A key part of this approach involved reducing the frustration frequently experienced by those who have to carry out highly repetitive rehabilitation exercises. Magicians and therapists interested in participating could obtain ‘The Project Magic Handbook’, which described a large number of magic tricks, and presented information on how to implement a successful therapeutic program (Kaufman, 2002). In 1982, the American Occupational Therapy Association formally supported the initiative, and Project Magic has been adopted by many practitioners.

In 1988, American magician Kevin Spencer helped to create a similar program after himself being involved in a serious car accident and suffering head and spinal cord injuries (Spencer, 2014). Spencer’s ‘Healing of Magic’ initiative involved magicians teaching simple magic tricks to promote patients’ physical and psychological wellbeing.  Emulating ‘Project Magic’, Spencer produced a manual and DVD containing appropriate magic tricks and instructional material (Spencer & Spencer, 2012).

In 2003, magician Michael Walton created a program entitled ‘Open Heart Magic’ to teach magic tricks to children in hospital (Hart & Walton, 2010). The work aims to boost self-esteem and provide a sense of mastery. Unlike many of the other magic-based programs, all potential performers are required to participate in a rigorous training program. Although the initiative runs in various locations, much of the activity takes places in Chicago, where over 100 ‘Hospital Magicians’ visit 10 hospitals and perform to over 10,000 children each year.

In 2007, American medical student and former professional magician David Elkin founded ‘MagicAid’ (Elkin & Pravder, 2018). This initiative involves training health practitioners to deliver one-on-one ‘magic therapy’ for paediatric patients and their families, and aims to help reduce the stress that children often experience in hospital. MagicAid is run by both medics and magicians (including the well-known magician Justin Willman) and has been successfully implemented at several medical facilities.

In Canada, the educational and therapeutic program ‘My Magic Hands’ is run by magician Julie Eng (executive director of the magic-based arts organization, Magicana). This program has been running for several years and has involved magicians working with occupational therapists to help children with a range of disabilities. Other aspects of the program have included work with at-risk children and those attending community centers in economically disadvantaged areas.

Most recently, a team of British occupational therapists have partnered with magicians to create ‘Breathe’—a program designed to help those suffering from childhood hemiplegia (Breathe, 2015). Children with hemiplegia often find it difficult to continually carry out the repetitive exercises needed to help combat their condition. Breathe aims to tackle this issue by turning these exercises into magic tricks. During the initiative, children typically participate in around 60 h of intensive therapy and top-up workshops. In addition, they can perform in a show attended by their friends and families.

### Educational magic-based interventions

Within a pedagogic context, one of the earliest recorded references dates back to the turn of the last century and is associated with a seminal book on Victorian conjuring entitled ‘Modern Magic’ (Hoffman, 1876). Written by lawyer and magician Angelo Lewis (using the pseudonym ‘Professor Hoffman’), Modern Magic aimed to teach readers how to perform magic, and was arguably the first book to provide a highly detailed description of the apparatus and methods used by magicians. In 1877 the book was published in Russia and included a foreword by Russian educator and scientist Julian Simashko (Fedorov, 2018). In keeping with the then popular notion of ‘rational recreation’ (the use of leisure time for self-improvement and self-enrichment) Simashko noted:

‘It doesn’t matter that a magic trick is not normally viewed as serious education, it helps to develop a quick eye and a sharp inquisitive mind. A trick, a puzzle, a logical analysis, or a mathematical theorem—everything is good if leads to this aim…. To perform just one or two magic tricks you’ll have to do some work: to understand the secret of the trick, to complete all necessary preparations, and to practice until your hand becomes more dexterous and flexible. We saw that children successfully overcame those difficulties, they eagerly grasped the problem and did the necessary preparation to achieve their goal, in short they are thinking’.

Subsequently a handful of magicians and educators have built on this notion, producing books on how to get children to perform magic in the classroom and develop key skills (see, e.g., Windley, 1976; McCormack, 1990; Kett, 2002).

In the late 1980s, therapist Sadie Broome developed ‘The Magic Kids’ program for students with behavioral and emotional disorders. Broome (1989) and Broome (1995) offered guidelines for making a magic-based intervention effective within a school-based setting, and provided a detailed description of the tricks and illusions used during the show. Similarly, Bowman authored several books containing tricks that can be easily mastered, and described the potential life lessons that can be gained from each trick (Bowman, 1986; Bowman, 2002; Bowman, 2004). 

In South Africa, ‘The College of Magic’ was created by David Gore in 1980, and provides a wide range of magic-based training courses for children. The college aims to create a positive and caring environment that helps children to build a range of life skills, along with eight specific ‘Star Qualities’ (honesty, respect, responsibility, initiative, excellence, empathy, humility and wonder).

In 2011, Kevin Spencer built on his ‘Healing of Magic’ initiative by launching ‘Hocus Focus’, an educational curriculum that involves magic-based lesson plans designed to promote students’ motivation and key learning skills, including individuals with learning and emotional challenges (Spencer, 2012). Most recently magician Michael Ammar has launched his ‘Discover Magic’ initiative in which children are taught tricks designed to develop eight traits including self confidence, creativity, preparation, communication skills, and resilience (Johnston, 2016).

### Potential benefits of magic-based interventions

As outlined in the previous section, magicians, medics, educators, psychologists and occupational therapists have described a wide range of benefits that might flow from learning to perform magic tricks. We have created the following chronology-based framework to organise these benefits, outlining the various stages involved in learning, practicing and performing a magic trick.

#### Lateral thinking and problem solving

The methods used in magic tricks are often surprisingly simple, and frequently involve some form of lateral thinking. Over time, magicians learn the general principles involved and can use these to create new tricks. It seems likely that learning magic will encourage creative problem solving and may even help people to find novel solutions to challenges in their own lives. Indeed, some writers have suggested that the secrets to magic tricks symbolize optimism because they show how seemingly complicated problems can have remarkably simple solutions.

#### Fostering trust

When someone is told how a magic trick is achieved they are being trusted with secret information, and it is hoped that they will not tell others the secret to a trick. This process may help people to understand the importance of trust and to foster a spirit of togetherness.

#### Storytelling and imagination

Creating magic effects involves putting together a narrative that is both entertaining, holds onlookers’ attention, and justifies the actions involved in the trick. This is likely to help promote a series of related skills, including imagination, storytelling, and emotional empathy.

#### Practical skills

Although many magic tricks can be performed with everyday objects, some involve creating objects that are either on display during a performance (known as ‘props’) or those that are hidden from the spectators (known as ‘gimmicks’). The construction of these objects may promote practical building skills and an understanding of how to work with a variety of materials.

#### Cognitive skills

Learning how to perform a magic trick usually requires following a series of instructions and a considerable amount of practice. Therefore, learning magic may promote several key cognitive skills, including concentration, preparedness, self-control and memory.

#### Motor skills

Many tricks involve manipulating objects in unusual ways. Learning those tricks may help to promote gross and fine motor skills, and eye-hand coordination. In addition, occupational therapy often involves highly repetitive actions; incorporating these movements into a magic trick may make the actions more enjoyable and meaningful.

#### Teamwork

Although people are able to learn tricks on their own, magic is often far from a solidity experience. Tricks can be taught in groups, people can work together to build props and stage a show, and magicians are frequently members of magic societies and clubs. So, learning magic may help to develop teamwork skills, including learning to give and receive constructive feedback, identifying individuals’ strengths, and helping to build community.

#### Interpersonal communication

As with any type of public presentation, the successful performance of a magic trick involves basic acting skills. However, magic brings several additional challenges, including often having to give clear instructions, controlling onlookers’ attention, overcoming possible nervousness caused by having to conceal certain actions, and finding acceptable ways of explaining why one cannot reveal the secret of the trick. Therefore, the performances may promote interpersonal skills, emotional regulation, respect, and self-presentation abilities.

#### Adaptability and resilience

Unlike many performances, the presentation of magic often involves considerable flexibility, as the magician adapts the trick to a particular situation or has to deal with an unexpected event. In addition, tricks do occasionally go wrong, or onlookers may correctly guess the secret of the trick. Therefore any good performer has to be able to cope with failure. These experiences may help build both adaptability and resilience.

#### Self-esteem and confidence

Performing magic tricks also promotes self-esteem as the performer can do something the audience cannot, and provides a sense of pleasure and mastery from creating an unusual and enjoyable experience for others.

### Evaluation of benefits of magic-based interventions

Several researchers have produced anecdotal evidence and case studies to support the claim that learning magic tricks promotes wellbeing (see, for example, Stenhouwer, 1983; Frith & Walker, 1983; Geens, 2005; Fisher & Fisher, 2007; Harte & Spencer, 2014). This section outlines work that has involved a more systematic evaluation of these programs, focusing on both physical and psychological benefits. This work has generally been reported only in specialist academic journals and publications produced by the magic community.

#### Physical benefits

Four studies have assessed whether teaching people magic tricks may help their physical wellbeing.

Sui & Sui (2007) describe a two-year project that involved delivering aspects of the ‘Healing Is Magic’ program within a social services organization in Hong Kong. Thirty staff taught magic to patients diagnosed with a range of serious mental illnesses, including schizophrenia and depression. Forty patients completed the ‘Purdue Pegboard’ (a standard test of manual dexterity and bimanual coordination) before and after participating in the magic-based intervention. The patients showed significant improvements both in the use of their left and right hands, and in their ability to use both hands together.

Green et al. (2013) carried out research as part of the Breathe initiative, assessing the level of hand and arm use among 23 hemiplegic children before and after a two-week course of magic-based therapy. Participants completed two questionnaires measuring spontaneous and daily use of their affected hand (‘The Assisting Hand Assessment’ and ‘The Children’s Hand Experience Questionnaire’) and a standardized test of their ability to grasp and release items (the Jebsen-Taylor Test of Hand Function). The children displayed significant improvements on all three measures, although their scores on The Assisting Hand Assessment returned to pre-test levels three months later.

As part of the same initiative, Weinstein et al. (2015) had 12 hemiplegic children undergo functional brain imaging (fMRI) before and after the Breathe intervention. The children showed significant increases in the level of activation in the affected hemisphere, and around half of them demonstrated a significant increase in white matter integrity in the corpus callosum and corticospinal tract. Additional work by Schertz et al. (2016) revealed that children with greater brain damage benefited most from the intervention.

#### Psychological benefits

Ten studies have assessed whether teaching people magic tricks may help their psychological wellbeing.

Lyons & Menolotto (1990) undertook a pilot study in which a magician and two occupational therapists taught magic tricks to seven psychiatric patients with a variety of conditions (including schizophrenia, recurrent paranoid psychosis and bipolar affective disorder). The magic-based intervention consisted of eight 90-minute, sessions, delivered over a nine-week period. The tricks were chosen from Copperfield’s ‘Project Magic’ and aimed to enhance patients’ social skills. Six patients completed a questionnaire about the benefits of learning magic, with the results showing that they found the experience highly sociable, refreshing, pleasant and meaningful. The authors note that the programme helped lend structure to large amounts of uncommitted time, and that a key part of its success involved discovering strengths rather than emphasising limitations.

Ezell & Klein-Ezell (2003) examined whether learning magic tricks helped boost the self-esteem of children with physical and psychological challenges. Ten university students taught magic tricks to 26 children with a range of conditions, including learning difficulties, behavioral disorders, and physical disabilities. Many of the tricks were selected from ‘Project Magic’ and the children were given the opportunity to rehearse in front of a mirror and also to perform for younger children. The children’s self-esteem (measured via ‘The Student Self-Concept Scale’) was significantly higher after the intervention.

Kwong (2007) taught magic tricks from the ‘Healing of Magic’ manual to 11 inpatients on a Neuro-Rehabilitation ward over several weeks. 5 participants completed pre and post questionnaires designed to measure their quality of life (EQ-5D), self-esteem (Rosenberg Self-Esteem Scale), and mood (Rand Health Survey). Scores on the EQ-5D and Self-Esteem Scale showed non-significant increases, and only one of the 8 sub-scales within the Rand Health Survey was significant (‘Energy/Fatigue’; *p* = 0.02).

Levin (2007) examined whether a magic-based intervention might boost the self-esteem and positive behavior of severely emotionally disturbed children. Nine participants (aged between 6 and 18) were taught magic tricks during six, one-hour, weekly sessions. Six children completed the Rosenberg Self-Esteem Scale pre- and post the intervention. Although the authors do not provide statistical data, they report that the children’s scores increased on 8 of the 10 scale items. The group also displayed several behavioral improvements, including a decrease in ‘boundary violations’ (mean of 20 violations per child to 7 per child), and ‘time-outs’ (mean of 13.67–5.17 per child).

Sui & Sui’s (2007) study, described above, also examined whether a magic-based intervention improved patients’ psychological wellbeing. Forty patients completed questionnaires designed to measure their happiness and general satisfaction (Personal Wellbeing Index) and self-confidence (Chinese General Self-Efficacy Scale). The researchers report significant increases on the Personal Wellbeing Index and on just one item of the General Self-efficacy Scale (the capability to identify multiple solutions to a problem). Patients indicated that their cognitive skills (including memory, concentration and ability to think rationally) had improved, and that the intervention boosted their confidence and acted as a catalyst for conversation. Interestingly, the researchers also noted that the patients became more presentable during performance.

Spencer (2012) conducted a study examining the psychological impact of the Hocus Focus programme in three schools, involving a total of 9 teachers and 76 students (aged between 12 and 14). The students were diagnosed with a range of issues, including Autism, Emotional Behavior Disorder, Learning Disability, ADHD, and Communication Difficulties. The teachers completed various observation checklists and surveys, and students completed both surveys and informal interviews. Qualitative data indicated that the majority of the students experienced improvements. The teachers reported that the intervention captured and held students’ attention, encouraged active participation, emphasized the importance of following directions, and encouraged students to help one another. The students appeared to show increases in concentration, and memory skills, self-determination and self- esteem, motivation and participation, leadership and socialization, peer relationships and collaboration.

As part of the research surrounding the Breathe initiative, 12 hemiplegic children completed a questionnaire-based measure of hopefulness (HOPE scale) both before and after learning to perform magic tricks (Green & Farquharson, 2013). The results showed a large (but non-significant) increase in hopefulness (*p* = 0.06, 2-tailed), and comments from parents reflected themes of empowerment and improved confidence.

Similarly, Hines et al. (2018) carried out semi-structured interviews with 29 parents of hemiplegic children who had undertaken the Breathe initiative. Three main benefits emerged: ‘It’s okay to be me’ (parents believing that their children experienced a strong sense of inclusion and insight), ‘the magic effect’ (valuing the novelty and challenge of performing magic tricks), and ‘I can do it’ (increased self-belief and a willingness to attempt challenging tasks). Overall, many parents believed that their children had gained a feeling of mastery, which had then helped them to develop a more autonomous approach to daily activities.

Bagienski (2016) examined whether performing magic elevated the performer’s mood. Around fifty University students were either taught to perform a simple magic trick or given techniques to boost their rapport skills. Each day participants performed the trick or used their rapport building techniques, assessed how successful they had been, and rated their positive and negative mood (PANAS scale). Nineteen participants provided enough data to be entered into the analysis. Contrary to the hypothesis, the affective states and success rates of participants who had learned the magic trick were not significantly different from those who had been taught rapport building skills.

Finally, Pravder et al. (2018) examined whether a magic-based intervention created by the MagicAid initiative helped reduce the psychological discomfort and anxiety of pediatric patients and their caregivers during hospitalization. Medical students at Stony Brook Children’s hospital were taught magic tricks, and then taught these tricks to patients aged 5–16 years. 101 patient-caregiver pairs were randomly assigned to receive either magic therapy or ‘standard services’ (which included pet therapy, art therapy and music therapy), and self-report anxiety levels were taken pre and post intervention (‘Facial Image Scale’, ‘Venham Picture Test’ and ‘Short State-Trait Anxiety Inventory’). Compared to participants who received the ‘standard services’, those in the magic-based intervention were significantly less anxious on post-test measures.

## Discussion

This paper has presented an overview of the many systematic programs that aim to promote wellbeing by teaching people how to perform magic tricks. These programs have been developed within both a health setting and an educational context, and date back to the turn of the twentieth century. Various authors have identified the key benefits that might flow from a magic-based intervention, including a boost in self-esteem, an increased feeling of mastery, and gains in motor skills. Some of the work supporting these benefits is anecdotal in nature, and involves either clinicians’ and educators’ first-hand experience or their descriptions of case-studies. A relatively small amount of work has adopted a more systematic approach to evaluation of the benefits of magic-based interventions. In terms of potential physical benefits, this research has mainly been carried out by those associated with the ’Breathe’ initiative, that focuses on helping hemiplegic children. Overall this work has reported highly positive findings, suggesting that a magic-based initiative can help boost the children’s motor skills and range of movement. Similar work examining the potential psychological benefits of magic-based interventions has assessed a range of factors, including general life-satisfaction, self-esteem and behavioural problems. Again, overall positive results have been reported.

Although promising, there are several reasons to treat the results from both sets of studies with caution. First, with the exception of two studies (Bagienski, 2016; Pravder et al., 2018) these studies have not employed a control group, and so it’s difficult to assess how the outcomes associated with learning magic tricks compare to either doing nothing or other interventions. Second, almost all of the studies have involved relatively small numbers of self-selected participants, and so it is unclear whether the findings apply to larger cohorts. Third, almost all of the studies have involved participants who are facing physical and psychological challenges, and so it is not yet clear whether magic-based intervention are beneficial to non-clinical populations (again, Bagienski (2016) is the only exception, and the null results reported in that study give reason to be cautious). Finally, the magic-based intervention employed in the studies differed on many dimensions (including one to one versus group delivery, the duration of the intervention, and the tricks involved) and so it is problematic to assess which parameters are maximally associated with successful outcomes.

It is hoped that future researchers will conduct additional work into the efficacy of magic-based interventions. Hopefully, this work will build upon the existing research base by the addition of control groups, using larger numbers of participants, exploring whether such interventions are effective for non-clinical populations, and exploring which aspects of the intervention are especially effective. In addition, whereas almost all of the previous work has aimed simply to document the potential impact of interventions, future research could adopt a more theoretical approach and explore why these interventions might be beneficial. This more theory-based work might, for instance, build on existing research suggesting that magic may have the potential to provoke curiosity (Subbotsky, 2010), create a certain form of wonder (Lamont, 2017), and enhance divergent thinking (Subbotsky, Hysted & Jones, 2010; Danek et al., 2014).

A large amount of research has examined the potential therapeutic impact of the performing arts and there are several journals are dedicated to the topic, including the *Journal of Music Therapy*, the *Journal of Poetry Therapy*, the *American Journal of Dance Therapy*, and *Drama Therapy Review*. In comparison, only a small amount of work has examined the relationship between magic-based interventions and wellbeing. This discrepancy could be due to several factors, including, for instance, performing arts researchers and practitioners tending not to have a background in magic, the naturally secretive nature of magicians, the erroneous notion that magic isn’t an art form, or the idea that performing even basic magic tricks involves considerable manual dexterity. Removing these potential barriers may encourage more performing arts researchers and practitioners to explore magic-based interventions. We believe that this would be highly desirable, in part because magic-based interventions have several practical advantages over other performing arts. For example, most people, including children, find the idea of being able to perform a magic trick an engaging, attractive and interesting proposition. This is especially the case at the moment, due to the success of several fantasy and magic-based books and films, such as ‘Harry Potter’ and ‘Lord of the Rings’. Second, many entry-level magic tricks can be learned quickly and require relatively modest skills. Third, a large number of tricks require inexpensive equipment and so are cost effective. Fourth, it is easy to create activities that can be tailored to specific abilities and situations, and can be used in both a one-to-one and small group setting. Fifth, magic allows people to practice on their own but also involves social interaction during a performance. Finally, magic tricks represent a highly structured form of interaction that can be used in everyday life to break the ice and build rapport with friends and strangers.

## Conclusions

The work into the efficacy of magic-based interventions has been reported in specialist academic journals and publications produced by the magic community. This paper aims to bring this literature to a wider audience and to review its findings. The literature suggests that learning to perform magic ticks can help promote both physical and psychological wellbeing. As is often the case in fledgling areas, the work suffers from various methodological issues that make it problematic to draw firm conclusions. In line with the magic-based interventions being reported, it is hoped that future researchers are able to make these problems disappear and, in doing so, discover how magic can help to produce some genuinely remarkable phenomena.

## Additional Information and Declarations

### Data Availability

The following information was supplied regarding data availability: It is a literature review, and so the study did not generate raw data.
